# The integration of chloroplast protein targeting with plant developmental and stress responses

**DOI:** 10.1186/s12915-017-0458-3

**Published:** 2017-12-07

**Authors:** Lynn G. L. Richardson, Rajneesh Singhal, Danny J. Schnell

**Affiliations:** Department of Plant Biology, Michigan State University, 612 Wilson Road, Room 166, East Lansing, MI 48824-1312 USA

## Abstract

The plastids, including chloroplasts, are a group of interrelated organelles that confer photoautotrophic growth and the unique metabolic capabilities that are characteristic of plant systems. Plastid biogenesis relies on the expression, import, and assembly of thousands of nuclear encoded preproteins. Plastid proteomes undergo rapid remodeling in response to developmental and environmental signals to generate functionally distinct plastid types in specific cells and tissues. In this review, we will highlight the central role of the plastid protein import system in regulating and coordinating the import of functionally related sets of preproteins that are required for plastid-type transitions and maintenance.

## Plastid protein import: from cytoplasm to organelle

The plastids are a structurally and functionally diverse group of inter-related organelles that are a hallmark feature of plants and algae. In addition to the reactions of photosynthesis in the chloroplasts of green tissues, the plastids confer a remarkable degree of metabolic diversity in all plant tissues. This includes basic components of amino acid and lipid metabolism, and specialized metabolism, including starch metabolism in roots and tubers by amyloplasts, carotenoid synthesis in ripening fruit and some floral tissues by chromoplasts, and the synthesis of tissue-specific phytohormones and key defense and signaling molecules in a variety of tissues. Plastids are essential organelles that are derived from undifferentiatied proplastids and undergo specific differentiation to fulfill specialized functions, depending on developmental changes or physiological and environmental conditions [1, 2]. Plastid differentiation also is reversible and different plastid types can rapidly interconvert depending on the metabolic demands of the cell or tissue type.

**Fig. 1.**
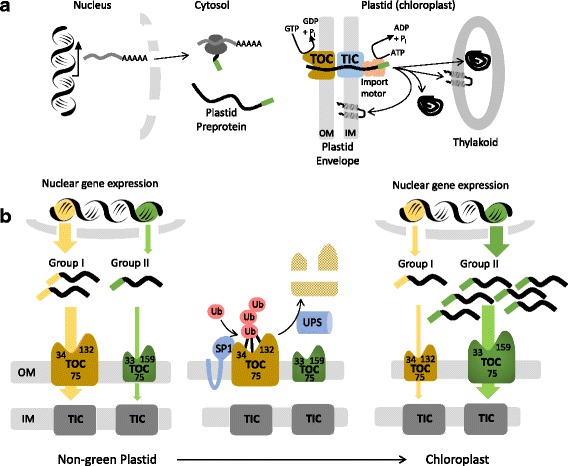
Plastid protein import and control of the import machinery by the ubiquitin-proteasome system. **a** The majority of plastid proteins are encoded in the nucleus and translated on cytosolic ribosomes. Plastid preproteins contain an N-terminal transit peptide that is necessary and sufficient to target proteins to the organelle. The transit peptide is recognized at the surface of the plastid by two GTPase receptors of the TOC complex (*brown*), Toc159 (*159*) and Toc33 (*33*), at the outer envelope membrane (*OM*). The receptors initiate membrane transport via a GTP-dependent switch, and the preprotein translocates through an associated β-barrel channel, Toc75 (*75*) of the TOC complex. Import occurs simultaneously across TOC and TIC (*blue*) and is driven by an ATP-dependent import-associated chaperone network, which constitutes the import motor (*orange*). The transit peptide is removed by the stromal processing peptidase upon import, and the chaperone network assists in folding and assembly of the newly imported proteins. Proteins destined for the inner envelope or thylakoid membranes are subsequently recognized by conserved sub-organellar targeting machineries. **b** Distinct TOC complexes (*brown* and *green*), defined by the presence of specific TOC GTPase receptors (e.g., Toc159/33 vs. Toc132/34) mediate import of specific classes of preproteins, thereby preventing competition for import between proteins from different functional or developmental-specific groups (e.g., Groups I and II) and providing a mechanism of selectively regulating their import. The turnover of TOC complexes plays a key role in plastid-type transitions, including the conversion from chemoautotrophic to photoautotrophic metabolism in seedlings. TOC complex turnover is controlled by the ubiquitin proteasome system (UPS) via an outer envelope-associated RING-type E3 ubiquitin ligase, SP1. This functions to balance the levels of specific TOC pathways with changes in the expression of specific classes of preproteins to maintain organelle homeostasis

Greater than 90% of plastid proteins (~2500 different proteins in *Arabidopsis thaliana*) are encoded in the cell nucleus and synthesized on free ribosomes in the cytoplasm as preproteins containing an N-terminal extension, called a transit peptide. The transit peptide functions as the targeting signal to direct the preprotein to receptors at the organelle surface. Transit peptide-receptor binding initiates import of the preprotien into the organelle from the cytoplasm (Fig. 1a). Protein import occurs through a complex protein targeting system that mediates the transport of preproteins across the plastid outer and inner envelope membranes and serves as the gateway for at least five other sub-organellar protein targeting pathways that are necessary to maintain internal membrane systems, such as the light-harvesting systems of the thylakoid membranes [1, 3–6]. The protein import system must adapt to facilitate the rapid turnover of the plastid proteome and the remodeling of internal membrane systems necessary for plastid-type transitions in response to the changes in gene expression that accompany tissue differentiation or adaptation [7–11].

A number of groups, including our own, have shown that the import of the majority of nucleus-encoded proteins into plastids is mediated by two multi-component transport systems at the double membrane envelope, referred to as the TOC (transocon at the outer membrane of chloroplasts) and TIC complexes (transocon at the inner membrane of chloroplasts) (Fig. 1a) [3, 4, 6, 12–14]. The intrinsic transit peptide of the nuclear-encoded plastid preprotein mediates import, and is recognized sequentially by TOC-TIC components and associated chaperones to drive selective, unidirectional transport through membrane channels at both envelope membranes [15–17]. The TOC-TIC system is aided by an additional array of molecular chaperones that facilitate targeting and membrane translocation of preproteins [18].

Traditionally considered a housekeeping function, it is now clear that protein import is a highly dynamic and regulated system, which adapts to accommodate the dramatic changes in the flux and profiles of imported proteins that occur during developmental and biogenetic events [6, 14]. This review will highlight recent studies that reveal 1) the diversity and regulation of the import apparatus that is required to mediate the import of different coordinately expressed proteins during developmental changes; 2) the concerted interactions of the TOC-TIC system with the chaperone and protein quality control networks in the cytoplasm, intermembrane space, and plastid stroma that maintain organelle proteostasis; and 3) the relationship of the protein import apparatus with sub-organellar targeting pathways that is essential to the organization and remodeling of organelle architecture.

## Diverse TOC complexes and the ubiquitin-proteasome system interact to maintain the balance of protein import during plastid biogenesis

The ability of plastids to undergo rapid functional diversification in different cell types requires a tight coordination between nuclear gene expression, translation, protein import, and assembly of organelle proteins [1]. For example, the light-induced transition of non-green plastids to photoautotrophic chloroplasts in green seedlings triggers the expression of hundreds of photosynthetic proteins that must be imported and assembled [2]. As a consequence, the preprotein targeting and import apparatus must dramatically increase its capacity to accommodate new sets of highly expressed proteins while maintaining the delivery and import of constitutively expressed plastid genes that are essential for basic organelle function.

Evidence is accumulating in support of the existence of distinct pathways for the import of specific classes of preproteins, thereby preventing competition for import between proteins from different functional or developmental-specific groups. The distinct pathways also provide a mechanism to selectively regulate the import of different functional groups of plastid preproteins (Fig. 1b). Studies indicate that the import of different preproteins varies between distinct plastid types or developmental stages of plant tissues [19, 20]. One detailed study identified at least three classes of preproteins whose import varies depending on the developmental stage of chloroplasts [20]. These include preproteins that are preferentially imported in newly developing chloroplasts (Group I), in mature chloroplasts (Group III), or those whose import efficiency is independent of the stage of plastid development (Group II). These data suggest that the import apparatus and transit peptides have coordinately evolved to optimize the import of different preproteins at distinct stages of organelle development [16, 17].

The functional diversity of transit peptides is consistent with the existence of functionally distinct TOC complexes and also supports the importance of multiple import pathways in plastid biogenesis [21–26]. Preproteins are recognized at the chloroplast surface by two related GTPase receptor families of the TOC complex, referred to as the Toc159 and Toc34 receptor families (the numbers refer to their predicted molecular sizes) [27–29]. We and others have proposed that Toc159 and Toc34 work coordinately to recognize the transit peptides of preproteins in the cytoplasm and initiate transport through an associated outer membrane channel, Toc75, via a GTP-regulated switch [6, 30–32]. Toc159 and Toc34 are encoded by small, differentially expressed gene families, and it has been demonstrated that the selectivity of TOC complexes for different preproteins in vitro and in vivo is determined by the GTPase receptor component of the TOC complex, primarily the specific Toc159 family member (Fig. 1b) [21, 25, 33]. In the *ppi2* mutant of *Arabidopsis thaliana* TOC159 (atToc159), the gene encoding the dominant Toc159 family member in the green tissues of *Arabidopsis*, plastids fail to develop into fully functional chloroplasts and the plants exhibit a pronounced albino phenotype [23]. The plants are unable to transition from chemoautotrophic to photoautotrophic growth, indicating that atTOC159 is required for the transition from non-green plastids to chloroplasts. Two other members of the Toc159 family, atToc132 and atToc120, are constitutively expressed in all tissues and appear to have overlapping transit peptide specificities [22, 26] that are distinct from atToc159 [25]. Their absence is lethal at an early stage of plant embryo development, and analysis of the atToc132 receptor suggests that it is required for the import of genes involved in basic plastid metabolism present in all tissues (e.g., amino acid and lipid synthesis) [22, 26]. The expression profiles of atToc159 and atToc120/132 correlate with the Group I and Group II classes of transit peptides, suggesting that they are primary determinants of distinct import pathways that are required for plastid maintenance and differentiation [17]. Detailed analyses of the transcriptome and proteome of *ppi2* plants suggest that the atToc159 pathway is involved in the biogenesis of a much broader spectrum of proteins than simply photosynthetic proteins, and that the contribution of distinct TOC complexes to plastid differentiation is complex and involves multiple import pathways [34]. Consistent with this conclusion, the Toc159 family members are expressed in all tissues examined, albeit at different absolute and relative levels [22, 26, 35, 36].

Despite the correlation between functionally differentiated classes of transit peptides and import receptors with distinct specificities, it remains unclear how a small number of import receptors (six identified to date) can accommodate the remarkable diversity of transit peptide sequences [37]. Li and Teng [17] have proposed a “multi-selection and multi-order” model for transit peptide organization to account for some of this functional diversity. They propose that transit peptides are comprised of sets of motifs that specify interactions with specific translocon components. The assembly of these motifs in a variety of combinations could then determine the plastid-type preferences for import and potentially determine the relative affinities and efficiencies of import by utilizing a fairly small number of import components.

The importance of distinct import pathways is highlighted by the discovery that regulated proteolysis via the cytoplasmic ubiquitin proteasome system (UPS) pathway controls the relative abundance of TOC complexes that are required for plastid-type transitions (Fig. 1b). Mutations in SP1, an integral outer membrane E3 ligase, disrupt the transition from etioplasts to chloroplasts in developing seedlings and chloroplasts to gerontoplasts in senescing plants [38]. Conversely, overexpression of SP1 promotes the etioplast to chloroplast transition and triggers early senescence. SP1 participates in the ubiquitination of both GTPase receptors and Toc75, and proteasome inhibitors inhibit their degradation [38]. Interestingly, SP1 expression and associated TOC turnover were increased under abiotic stress conditions that increased chloroplast ROS production [39]. These observations led to the hypothesis that regulated proteolysis is required for selective turnover of specific TOC complexes, thereby altering the balance of import pathways that are required for plastid-type transitions or in response to cell stress [40–42].

## Chaperone and quality control networks monitor preprotein competence during protein import

**Fig. 2.**
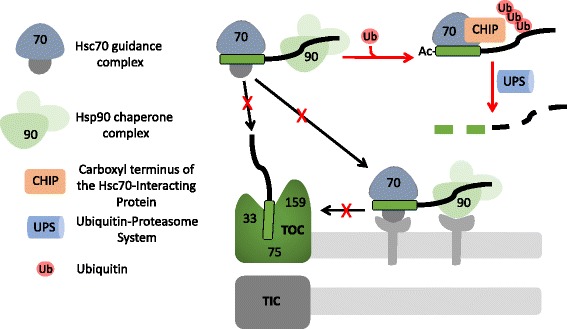
Targeting of preproteins to the TOC complex is monitored by the ubiquitin proteasome system in the cytosol. Preproteins are targeted to the TOC complex post-translationally as unfolded polypeptides, and cells must monitor protein import to avoid the toxic accumulation of mis-sorted or misfolded preproteins in the cytosol. This is particularly critical during plastid developmental transitions when TOC complexes are turned over, or under stress conditions when import is inhibited. Cytosolic Hsp70 (*blue*) and Hsp90 (*green*) chaperones have been implicated as components of targeting complexes to assist preprotein transit through the cytoplasm *en route* to their interaction with the TOC receptors (*black arrows*). When import of preproteins is inhibited (*red X*), an Hsp70 isoform, Hsc70-4, functions in conjunction with the cytosolic E3 ubiquitin ligase, CHIP (*orange*), to target misfolded or mis-sorted preproteins for degradation by the cytosolic ubiquitin-proteasome system (*UPS*; *red arrow*). N-terminal acetylation is prevalent under conditions that result in the accumulation of preproteins in the cytosol, suggesting that this might serve as a marker for UPS-mediated degradation

The ability of cells to synchronize TOC complex turnover and reorganization with the changes in import substrate profiles driven by developmental events or under stress conditions requires robust mechanisms to avoid mis-targeting and/or misfolding of preproteins during transport. For example, preprotein synthesis during chloroplast development reaches > 25% of total cellular gene expression as cells build the elaborate apparatus necessary for photosynthesis, putting a tremendous pressure on cellular proteostasis [2]. Preproteins are targeted to the TOC complex post-translationally as unfolded polypeptides, and there is evidence that the cytoplasm possesses a monitoring system to avoid the toxic accumulation of mis-sorted or misfolded chloroplast preproteins. Cytosolic Hsp70 and Hsp90 chaperones have been implicated as components of targeting complexes to assist preprotein transit through the cytoplasm *en route* to their interaction with the TOC-TIC system (Fig. 2) [43–46].

There is also evidence to suggest that components of the cytosolic chaperone complexes work in conjunction with a cytosolic quality control system to ensure that plastid preproteins do not accumulate in the cytoplasm. An Hsp70 isoform, Hsc70-4, functions in conjunction with the cytosolic E3 ubiquitin ligase, CHIP, to target misfolded or mis-sorted preproteins for degradation by the cytosolic UPS (Fig. 2) [47, 48]. Mutations in TOC components that decrease protein import efficiency result in the accumulation of preproteins in the cytoplasm [26, 36, 49] and induce the expression of Hsc70-4 [47]. Likewise, reduction in Hsc70-4 expression results in the appearance of cytosolic preproteins [47]. Specific recognition of cytosolic preproteins by Hsc70-4 for degradation appears to involve the transit peptide [47] and is correlated with detectable N-acetylation of cytosolic preproteins [34], suggesting a possible mechanism for the detection and marking of preproteins for selective degradation. It is not entirely clear how the quality control system distinguishes preproteins that are on a productive import route from those that are accumulating or import incompetent. However, the mechanism likely involves the probability of being detected by the quality control system during the transit time from the completion of protein synthesis to binding of the transit peptide at the TOC complex. Under optimal conditions, preprotein transit time would be short, reducing the probability of recognition by Hsc70-4/CHIP in the cytoplasm.

**Fig. 3.**
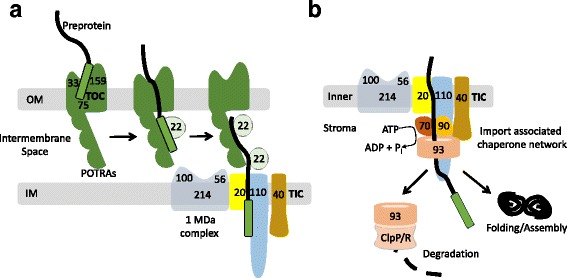
Chaperone systems in the intermembrane space and stroma assist cooperation between TOC and TIC and provide the driving force for import. **a** Import across the outer and inner envelope membranes (*OM* and *IM*) through TOC and TIC is coupled to provide direct targeting from the cytosol to the plastid stroma. Toc75 (*75*), the major membrane channel of the TOC complex (*green*), contains three polypeptide transport associated domains (*POTRAs*) that bind to preproteins in the intermembrane space as they emerge across the outer envelope. The POTRAs and Tic22 (*light green*), an intermembrane space chaperone, work together to ensure that preproteins do not misfold in the intermembrane space and assist in hand-off to the TIC machinery. In some species, preprotein targeting to the TIC system is facilitated by a 1 MDa complex at the inner membrane (*gray*) that includes Tic56 (*56*), Tic100 (*100*) and Tic214 (*214*). Tic20 (*20*), Tic110 (*110*), and Tic40 (*40*) are major components of the translocation machinery at the inner membrane. **b** Membrane translocation is driven by an import-associated chaperone network, containing cpHsp70 (70), cpHsp90 (90), and Hsp93/ClpC (93), which assemble at the site of import by the coordinate actions of Tic110 (*110*) and Tic40 (*40*). This chaperone network functions as an ATP-dependent import motor and may assist in folding and assembly of newly imported proteins. Recent evidence also suggests that Hsp93/ClpC is associated with the ClpP/R protease (ClpP/R), leading to the hypothesis that the Clp complex functions as a quality control system to degrade newly imported proteins that are orphaned or misfolded

Groups including our own have shown that, following GTP-dependent recognition by the TOC receptors, the first committed step of preprotein import corresponds to the ATP-dependent insertion of the preprotein into the TOC complex [50–53]. At this stage, the preprotein must traverse TOC and engage the TIC complex while avoiding mis-sorting in the intermembrane space. The events that occur in the intermembrane space leading to interaction of the precursor with the inner membrane translocon are not entirely clear, but recent data suggest that multiple components in the intermembrane space cooperate to ensure that preproteins do not get sidetracked *en route* to the plastid interior (Fig. 3). The first critical interactions in the intermembrane space occur between the transit peptide and the translocation channel, Toc75 (Fig. 3a). In addition to a β-barrel membrane channel domain that serves as the conduit for preprotein translocation across the outer membrane, Toc75 also contains three soluble POlypeptide TRansport Associated (POTRA) domains oriented toward the intermembrane space [54, 55]. We have shown that the POTRA domains are essential for the early binding stages of import, and that they possess preprotein binding and molecular chaperone activities [55, 56]. These observations suggest that the POTRA domains act as a docking site to receive the preprotein as it emerges through the outer membrane channel and provide a chaperone activity to prevent it from misfolding in the intermembrane space.

An additional intermembrane space protein, Tic22, crosslinks with preproteins and directly interacts with chloroplast preproteins in vitro [55, 57]. The Tic22 orthologue from the apicomplexans *Plasmodium falciparum* and *Toxoplasma gondii* display chaperone activity in vitro [58]. Therefore, it has been proposed that Tic22 acts as a chaperone in the intermembrane space to facilitate the continuity of precursor translocation between the TOC and TIC channels (Fig. 3a). Plants expressing POTRA-deleted versions of Toc75 showed a dramatic increase in Tic22 levels, and the POTRA domains of Toc75 interact with Tic22 in vitro, suggesting a functional link between these intermembrane space components [55]. The crystal structure of the Toc75 POTRA domains was recently solved and reveals features unique to Toc75 compared to related members of the Omp85 family from bacteria [56, 59, 60]. POTRA2 and 3 (the two β-barrel-adjacent POTRAs) are critical for binding precursors and in vitro chaperone activity [56], suggesting that together the POTRA domains of Toc75 and Tic22 act as a chaperone system for preproteins as they traverse the intermembrane space and engage the TIC complex.

To gain access to the chloroplast stroma, precursors also must be translocated across the chloroplast inner envelope, requiring the establishment of a link between the TOC and TIC complexes. Strong evidence indicates that Tic20, a multi-spanning membrane protein at the inner membrane, is a key link between TOC and TIC and likely forms the channel in the inner envelope [57, 61–63]. A second protein with similarity to Tic20, Tic21, might also participate at this stage [62, 64]. In *Arabidopsis*, Tic20 assembles into a “1 MDa complex” containing Tic100 and Tic56, which are both localized to the intermembrane space, and Tic214, a large membrane protein encoded by the chloroplast genome [61]. The 1 MDa complex interacts with preproteins at an intermediate stage in import, suggesting that it might facilitate the handoff of the preprotein from the Toc75-Tic22 complex to the Tic20 channel (Fig. 3a) [61, 65, 66]. Although Tic20 is conserved in all plant genomes examined, Tic214, Tic100, and Tic56 are absent from the grasses, suggesting that TIC architecture has diverged in some plant lineages.

## The import-associated chaperone network in the stroma functions as an import motor and quality control system

Although the precise nature of the membrane translocation events at the TIC complex remain to be defined, preproteins have been shown to interact with components of a stromal import-associated chaperone network immediately upon exposure to the stroma (Fig. 3b). In addition to the 1MDa complex containing Tic56, Tic100, and Tic214, numerous groups, including our own, have shown that Tic20 also interacts with a large membrane-bound protein, Tic110 [57, 62, 67–70]. Tic110 has a large C-terminal soluble domain protruding into the stroma that acts as a scaffold for chaperone binding [67–69, 71, 72], and it associates with a co-chaperone protein, Tic40, which is embedded in the inner envelope by a single TMD [73, 74]. Tic110 and Tic40 are responsible for assembling the chaperone network, including chloroplast Hsp70, Hsp90, and Hsp93/ClpC, at the site of import to facilitate membrane translocation, folding or suborganellar targeting of preproteins (Fig. 3b).

A primary function of the import-associated chaperone network is to bind preproteins as they emerge from the TIC channel and provide the motor for translocation across the envelope membranes (Fig. 3b) [18, 75]. The relative contributions of the three chaperones to the import motor or downstream chaperone activities has not been completely resolved, but all three chaperones appear to play essential roles in the later stages of import [76–79]. The transit peptide appears to initially dock at a site near the membrane on Tic110 [67, 80]. This interaction facilitates the association of Tic110 with Hsp93/ClpC, a AAA^+^-ATPase chaperone family member [81–85], and results in hand-off of the preprotein to the chaperone through the activity of Tic40 [73]. Chloroplast Hsp70 and Hsp90 also associate with this complex via interactions with Tic110 [77, 79]. Hsp70 homologs are well established to function in protein translocation in other organelles (mitochondria and the endoplasmic reticulum), and *Arabidopsis* knockouts of each of two chloroplast Hsp70 homologs show import defects in vitro [79]. Chloroplast Hsp70 immunoprecipitates with preproteins and other TOC/TIC components during the late stages of import [79]. Interestingly, the expression of chloroplast Hsp70 mutants with altered ATPase activities in the moss *Physcomitrella* cause corresponding changes in the ATP-dependence of protein translocation across the envelope, providing strong evidence that Hsp70 plays a major role in the translocation motor [79, 86]. Many transit peptides also possess Hsp70-binding motifs that are important for protein import [15, 87], further supporting the central role of both cytosolic and stromal Hsp70s in preprotein targeting and membrane translocation. Hsp90 (Hsp90C) was more recently discovered as an import associated chaperone [25, 88]. We showed that Hsp90C associates with TIC components, and that Hsp90C inhibitors block the later stages of import, consistent with a role as part of the import motor [25].

The complexity of the plastid import-associated chaperone network raises interesting questions about other potential functions for this complex downstream of import. This includes a possible quality control function to monitor the folding, assembly, or suborganellar targeting of newly imported proteins (Fig. 3b). While the majority of chloroplast Hsp93/ClpC exists in the stroma where it associates with the ClpP/R core as part of the Clp protease complex [89–91], a significant proportion of Hsp93/ClpC is associated with the envelope through an interaction with Tic110 [73, 92, 93]. Evidence suggests that the envelope-associated Hsp93/ClpC is also part of an intact Clp protease complex [18, 93]. These results suggest that envelope-associated Hsp93/ClpC might also function in degradation of preproteins shortly after import, either to regulate their levels within the chloroplast or to serve as an import-associated quality control mechanism (Fig. 3b).

## Cooperation between import and suborganellar targeting machineries optimizes suborganellar targeting

**Fig. 4.**
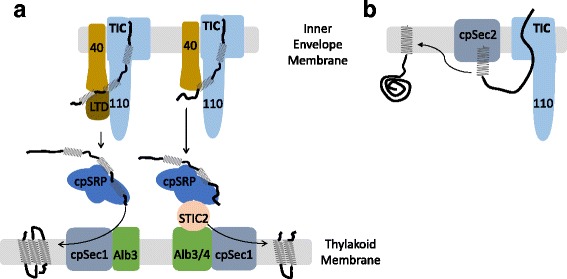
The TIC complex interacts with factors that facilitate the targeting of proteins to the inner envelope and thylakoid membranes to avoid potential mis-sorting of hydrophobic membrane proteins to the stroma. **a** At least five mechanisms conserved from the original bacterial endosymbiont exist for targeting proteins to the internal thylakoids of chloroplasts. The cpSRP system mediates the targeting of abundant thylakoid membrane proteins, including the light harvesting complex proteins. Genetic and biochemical evidence has identified a novel factor, LHCP translocation defect (LTD) protein, that docks at the inner membrane via an interaction with TIC components, Tic40 and Tic110, and also binds to cpSRP. LTD is proposed to facilitate the passage of LHCP proteins from the import apparatus to the cpSRP4 for delivery to the thylakoid Sec translocase (cpSec1). In a related pathway, Albino4 (ALB4), a homologue of ALB3, and STIC2, a homologue of bacterial YbaB, function by linking TIC and cpSRP to facilitate the targeting of a subset of proteins other than LHCPs to the thylakoid membrane. **b** Inner envelope membrane proteins are integrated into the membrane by a stop-transfer mechanism directly via the TIC channel or following import via a cpSec2 system (post-import/conservative mechanism) that catalyzes membrane protein integration from the stroma. For proteins using the post-import/conservative mechanism, the TIC complex and the cpSec2 system cooperate to allow protein import and membrane integration to proceed simultaneously to facilitate targeting to the inner membrane

For several hundred chloroplast proteins, import across the envelope membranes is only the first step in targeting to their proper location. Recent studies suggest that the TIC machinery also interfaces with factors that facilitate suborganellar targeting to the thylakoid and inner membranes to avoid mis-sorting to the stroma (Fig. 4). The thylakoid membranes possess multiple protein targeting pathways that are conserved from bacteria [5]. This includes the chloroplast signal recognition particle pathway or cpSRP/ALB3 system, composed of the cpSRP54 GTPase, the cpSRP43 chaperone, the cpFtsY receptor, and the Albino3 (ALB3) insertase, a protein related to the bacterial YidC and mitochondrial Oxa1 integrases [94–99]. The cpSRP pathway is responsible for delivery of the highly abundant light harvesting complex proteins (LHCP) to the chloroplast Alb3/cpSec1 translocase at the thylakoid [100, 101]. A recent genetic study identified a novel factor, LHCP translocation defect (LTD) protein, that docks at the inner membrane and is required for LHCP biogenesis [102]. LTD mutants are pale and defective in LHCP import and thylakoid targeting. LTD interacts with LHCP proteins while they are in the TIC channel via an interaction with Tic40 and Tic110, and also binds to cpSRP [102]. On this basis, LTD is proposed to facilitate the passage of LHCP proteins from the import apparatus to the cpSRP4 for delivery to the thylakoid Sec translocase (cpSec1) (Fig. 4a). Another genetic screen identified Albino4 (ALB4), a homologue of ALB3, and STIC2, a homologue of bacterial YbaB, as suppressors of the pale-yellow phenotype of a Tic40 mutant in *Arabidopsis* [103–106]. Both proteins appear to function in a targeting pathway involving cpSRP54 and its receptor cpFtsY for a subset of thylakoid proteins other than LHCPs [103–106]. Taken together, the genetic and physical interactions described for components of the TIC and the cpSRP pathway provide compelling evidence in support of close cooperation between the import machinery and the thylakoid targeting machineries (Fig. 4a).

The nuclear and plastid genomes also encode proteins destined to the inner envelope membrane [88, 107, 108] and evidence suggests a link between import of a subset of these proteins and their integration into the inner membrane (Fig. 4b). The inner membrane has its own conserved SEC system, designated cpSec2, which appears to mediate the integration of proteins following synthesis in the stroma or after import via TOC-TIC [109, 110]. Our own examination of the post-import/conservative pathway demonstrated that a single-pass inner membrane protein with an N-terminal transmembrane helix could integrate while still in the process of import through TOC-TIC [88]. In addition, Li and colleagues showed that FtsH12, an integral inner membrane protein, is targeted in a coupled import-integration manner, and they proposed that its two transmembrane domains and the intervening loop are inserted into the inner membrane from the stroma side via cpSec2 as the remainder of the polypeptide is imported through TOC-TIC [111]. This model is analogous to the insertion of mitochondrial Mdl1, in which the TIM23 machinery and the Oxa1 translocase cooperate to insert the protein at the inner mitochondrial membrane [112]. It will be interesting to see if this cooperation between the import and insertion pathways is a general feature for the insertion of inner membrane proteins. Regardless, it is clear from studies of targeting to the thylakoid and chloroplast inner envelope that mechanisms have evolved to minimize the probability of aggregation and mis-sorting of hydrophobic membrane proteins as they transit the stroma *en route* to their final destination.

## Protein import as a key control point in development and environmental responses

It is now clear from recent studies that the plastid protein import machinery functions both as an essential organellar protein targeting system and a key control point in remodeling and balancing the plastid proteome during developmental transitions and stress. This opens an exciting new focus on better understanding how the regulation and monitoring of protein import fits into the transcriptional and translational regulatory networks that contribute to organelle biogenesis, developmental transitions, and stress responses in plant systems.
